# Rockburst Disaster Prediction of Isolated Coal Pillar by Electromagnetic Radiation Based on Frictional Effect

**DOI:** 10.1155/2014/814050

**Published:** 2014-06-18

**Authors:** Tongbin Zhao, Yanchun Yin, Fukun Xiao, Yunliang Tan, Jianchao Zou

**Affiliations:** ^1^State Key Laboratory Breeding Base for Mining Disaster Prevention and Control, Shandong University of Science and Technology, Qingdao, Shandong 266590, China; ^2^Heilongjiang Ground Pressure & Gas Control in Deep Mining Key Lab, Heilongjiang University of Science and Technology, Harbin, Heilongjiang 150027, China; ^3^College of Mining and Safety Engineering, Shandong University of Science and Technology, Qingdao, Shandong 266590, China

## Abstract

Based on the understanding that charges generated during coal cracking are due to coal particle friction, a microstructure model was developed by considering four different variation laws of friction coefficient. Firstly, the frictional energy release of coal sample during uniaxial compressive tests was investigated and discussed. Then electromagnetic radiation method was used to predict the potential rockburst disaster in isolated coal pillar mining face, Muchengjian Colliery. The results indicate that the friction coefficient of coal particles decreases linearly with the increase of axial loading force. In predicting the strain-type rockburst, the high stress state of coal must be closely monitored. Field monitoring shows that electromagnetic radiation signal became abnormal before the occurrence of rockburst during isolated coal pillar mining. Furthermore, rockburst tends to occur at the early and ending stages of isolated coal pillar extraction. Mine-site investigation shows the occurrence zone of rockburst is consistent with the prediction, proving the reliability of the electromagnetic radiation method to predict strain-type rockburst disaster.

## 1. Introduction

The electromagnetic radiation occurs with the crack of rock under stress loading. The emission of this energy signal has been taken as the early warning of seismic activities [1–3]. In recent years, significant progress has been achieved in the aspects of physical mechanism, signal characteristics, early warning identification, and monitoring equipment of electromagnetic radiation. So far, it has been successfully applied to analyze regional high stress and monitor engineering disasters, such as rockburst and coal and gas burst. Typical work can be seen from the research work of He et al. and Wang et al. [4–6]. In uniaxial compressive tests, the coal sample fails due to aggressively developed cracks caused by the increase of uniaxial compressive stress. The crack growth and the emergence of new cracks reflect the sudden release of accumulated strain energy. From this perspective, the failure of coal can be seen as the complicated process of the growth of existing crack and emergence of new cracks. Laboratory study shows that charges generated from particle friction contribute to the occurrence of electromagnetic radiation for coal under axial loading [7].

The macro- and microfriction studies are an important part of rock mechanics. Since 1950s, the studies of frictional effect mainly concentrated on the mechanical interface behavior of rock joint or fault. The studies of China University of Mining Technology significantly advanced the understanding of electromagnetic radiation caused by friction of coal [7, 8]. The study of frictional effect among damaged coal particles can accurately reveal the mechanism of electromagnetic radiation. However, due to the limitation of experimental methods and equipment, the physical study on the friction among coal particles on the microscale is difficult. Instead, numerical experiments can be an effective way to bridge this gap.

The aim of this study is to provide theoretical guidance and foundations for the application of electromagnetic radiation method in predicting rockburst of coal mines. In detail, starting from viewport that friction among coal particles can generate charges and hence emit electromagnetic signals, the release of frictional energy during uniaxial compressive loading was investigated using the microparticle flow code—PFC. Later on, the prediction accuracy of electromagnetic radiation was estimated by applying it to predict the rockburst during isolated coal pillar extraction.

## 2. Frictional Energy Release of Coal in Loading

### 2.1. Friction Law among Coal Particles

**Figure 1:**
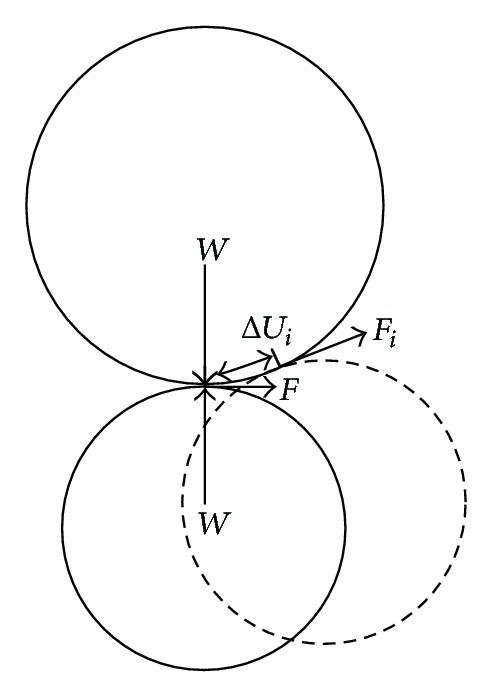
Friction between particle balls.

The coal is the aggregation of mineral particles and its internal friction can be considered as the relative slip among particles. Assuming that two particle balls are in contact at one point under the action of force *W* normal to contact plane (see Figure 1), the shear force *F* required for relative slip between two balls can be given by

(1) $$\begin{array}{c} F=\mu W, \end{array}$$

where *W* is normal force between coal particles, *F* is the shear force resulting in relative slip among particles, and *μ* is friction coefficient.

Amonton law states that friction coefficient *μ* only relates with the material, roughness, and state of contact plane, irrespective of normal force *W*. However, for coal, it has been proved that its friction coefficient *μ* is not invariable but changes with the normal force *W*; that is, *μ* = *f*(*W*). In general, it decreases with the increase in normal force *W* [9–12].

The action of shear force on particle yields relative slip. The energy dissipated can be defined as frictional energy. If the number of contact points within a coal sample is *m* and the number of damaged contact points is *n* under external loading, the dissipated frictional energy at the instant *t* can be calculated by

(2) $$\begin{array}{c} E_{f}=\overset{n}{\underset{i=1}{\sum }}F_{i}\Delta U_{i}, \end{array}$$

where *F*_*i*_ is shear force at the *i*th contact point, Δ*U*_*i*_ is the resultant slip displacement at the *i*th damaged contact point comparative to the state at last considered moment *t* − Δ*t*, and *E*_*f*_ is the released frictional energy at the instant *t*.

Equations (1) and (2) indicate that the released frictional energy correlates with normal force of particles, slip distance, and intrinsic property of contact point. The *W* and Δ*U*_*i*_ are easy to measure; however, the friction coefficient *μ* is comparatively difficult to determine.

### 2.2. Micromodel Development of Coal Particle Flow

**Figure 2:**
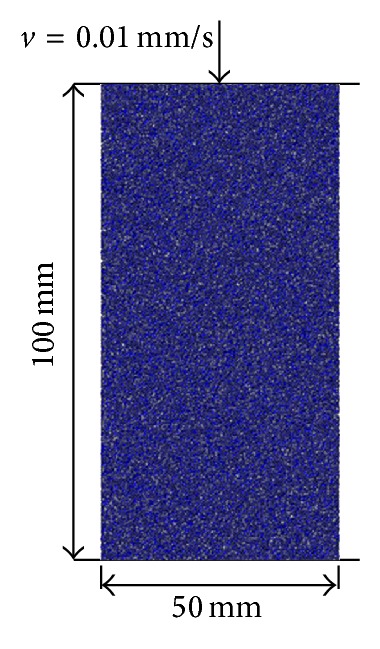
Generated model for uniaxial compressive test.

The model of uniaxial compressive tests was developed using the PFC and shown in Figure 2. The dimension of the model is 100 × 50 mm. In simulation, the axial loading was realized by applying a velocity of 0.01 mm/s at the top of the developed model. The physical and mechanical parameters of the model are summarized in Table 1. In computation during uniaxial loading, the stress, strain, crack, and frictional energy are monitored using in-built fish programming language.

**Figure 3:**
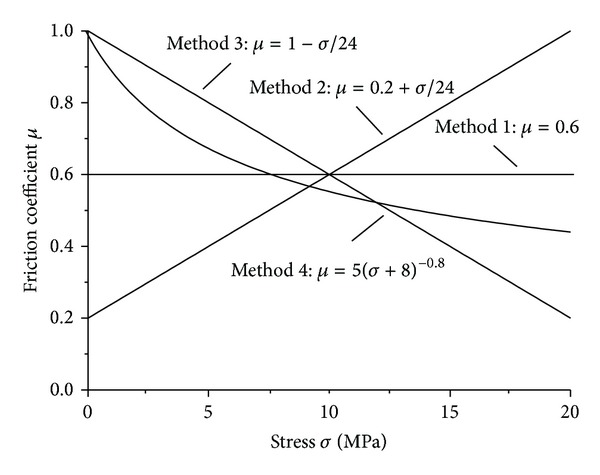
Choosing methods of the friction coefficient.

In order to study the influence of the friction coefficient on the frictional energy release before peak strength of coal sample, four different variation laws of friction coefficient were designed in the numerical modeling, where the mathematical expectation is 0.6: (1) method 1—the friction coefficient is a constant; (2) method 2—the friction coefficient increases linearly with the increase of axial loading; (3) method 3—the friction coefficient decreases linearly with the increase of axial loading, and (4) method 4—the friction coefficient decreases exponentially with the increase of axial loading.  Figure 3 shows these four change profiles of friction coefficient with the increase in axial loading.

### 2.3. Results Analysis

**Figure 4:**
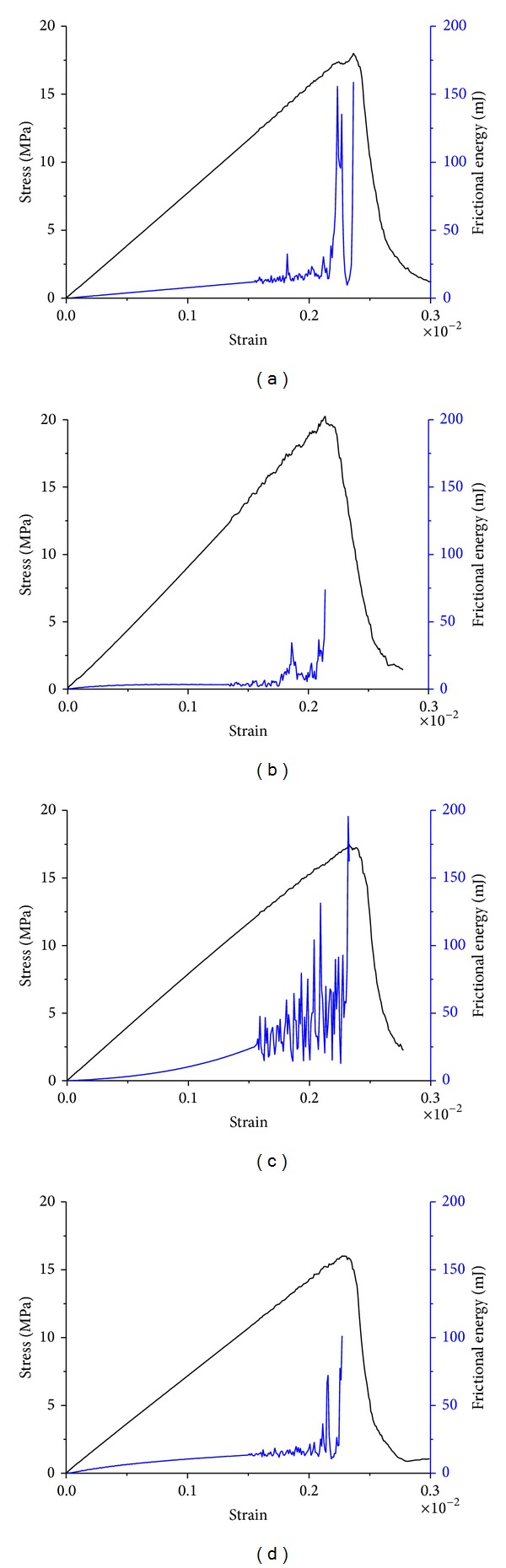
Frictional energy and stress curves of different methods. (a) Method 1; (b) method 2; (c) method 3; and (d) method 4.

**Figure 5:**
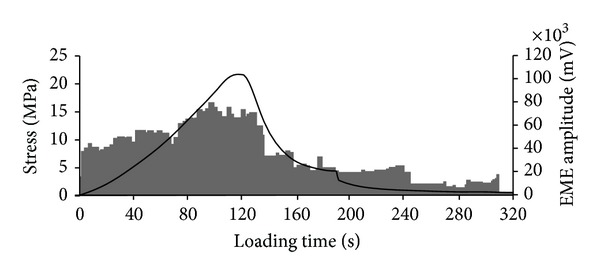
Curves of stress and electromagnetic amplitude.

Figure 4 shows the computerized results using different friction coefficient variation laws. At the comparative low stress-strain phase, the monotonous increase trend of released frictional energy is attributed to particle compaction or friction resistance between particles and boundary. It is not within the scope of this study and is not discussed further. When the accumulated deformation of coal sample approached a certain value, the shear slip among internal particles of coal caused the release of frictional energy and the amount of released frictional energy increased before meeting the peak strength of coal sample. By comparison, it can be seen from Figure 4 that the frictional energy of method 3 generates constantly and obviously in the loading process, which is in better accordance with the laboratory test result of electromagnetic radiation (Figure 5). Hence, the correct characterization of friction coefficient is crucial for accurate description of electromagnetic radiation energy caused by friction.

**Figure 6:**
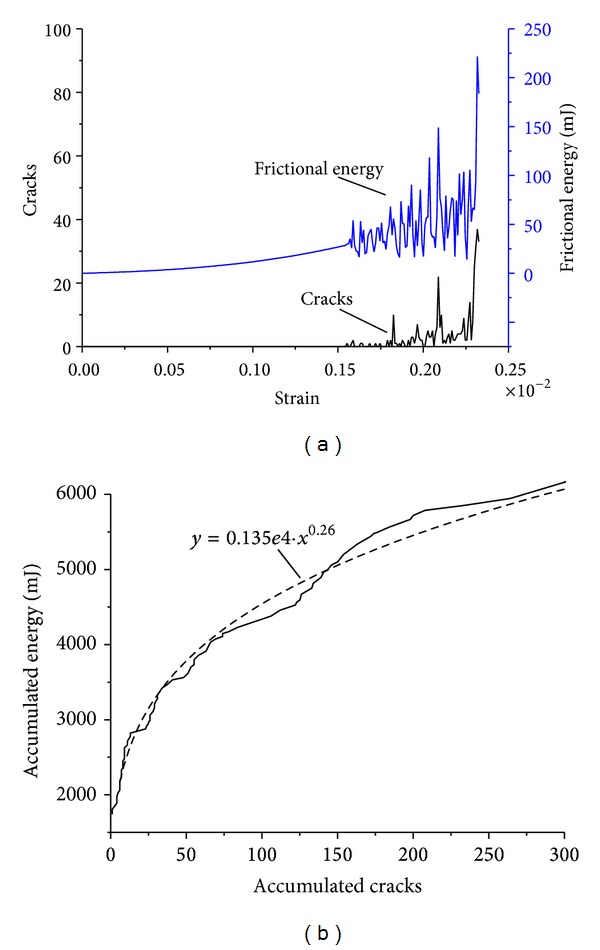
Relation curves of frictional energy and cracks. (a) Transient energy and cracks; (b) accumulated energy and cracks.

Using the computation result of method 3, the internal relation between frictional energy and cohesive damage among coal particles is discussed here (Figure 6). The release of frictional energy and the emergence of crack occurred at the same time, indicating that the energy of electromagnetic radiation comes from the damage slip among coal particles. Before the peak strength, the released frictional energy increased with the crack, and the release of accumulated frictional energy is exponentially proportional to the accumulated cracks. Under the control of decreasing friction coefficient, the release of frictional energy becomes slow before failure of the coal sample. Hence, the computational result of method 3 is consistent with laboratory electromagnetic radiation experiment during uniaxial compressive loading on coal samples. This provides a more proper explanation for application of electromagnetic radiation method in predicting high stress state of coal seam.

## 3. Mechanism of Electromagnetic Radiation in Predicting Strain-Type Rockburst

### 3.1. Electromagnetic Radiation Characteristics of Coal under Compressive Loading

Xiao et al. [13] recorded the electromagnetic radiation signal while conducting uniaxial compressive test on the coal sample and established the correlation between the compressive stress and the intensity of electromagnetic radiation as shown in Figure 5. It can be seen that at the initial stage of axial loading, the amplitude of the electromagnetic radiation signal increases with the loading before approaching the peak strength of coal sample. However, after reaching peak strength of coal sample, the signal amplitude decreases with further increase in axial loading.

On the microscale, these generated charges during coal cracking come from the charge separation. The charges from the electric double layer cannot disappear in time during the coal cracking and separated subsequently. At the loading phase before reaching peak strength of coal sample, the dominant part of the electric charges is generated by coal particle friction and the captured electromagnetic signal is very strong. Hence, frictional effect can reasonably explain this resultant electromagnetic radiation phenomenon.

### 3.2. Electromagnetic Radiation Method in Monitoring Stress of Surrounding Rock

The electromagnetic radiation monitoring system mainly consists of computer, receiving transducer, transform port, and connecting cable. It has the characteristics of noncontact, regional, continuous monitoring. The main monitoring indicators are amplitude intensity and pulse number.

**Figure 7:**
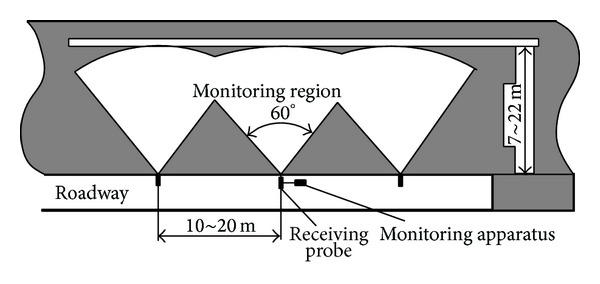
Monitoring region of electromagnetic radiation [7].

In application to monitor electromagnetic radiation signals of country rock in mining, noncontactable inductance type wideband directional receiving probe is usually used. The distance between the monitoring apparatus and the targeted mining wall is usually set in the range of 0.6–1.0 m and the internal among monitoring points is approximately 10 m. The effectiveness monitoring depth can be 7–22 m within the coal ore. The layout of the electromagnetic radiation monitoring positions is shown in Figure 7. The signals of electromagnetic radiation were recorded according to the number sequence of monitoring positions. The monitoring time for each point is 180 s.

### 3.3. Mechanism of Electromagnetic Radiation Method to Predict Strain-Type Rockburst

The rockburst has been one of the major engineering disasters perplexing the coal mining industry. There are still some disagreements on its deciding factor, physical mechanism, and prediction techniques [14–17]. Recently, the 973 research team on the project of “study on dynamic disaster mechanism and prevention of coal in deep mining” categorized the rockburst into three types: strain-type, slip-type, and mix-type. This categorization is of more significance for rockburst prediction and prevention.

**Figure 8:**
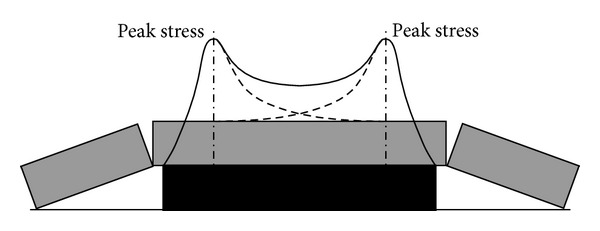
Abutment pressure distribution of the coal pillar.

The radical reason for the strain-type rockburst is due to high stress concentration caused by mining activities. Also, the strength of coal seam is usually lower than surrounding rock and the exposed working face also provides the essential space of such energy release. With the advance of mining extraction, elastic strain energy gradually accumulates in front coal body of mining face. When such accumulated elastic energy reaches its critical value, further engineering disturbance will trigger rockburst [18, 19]. Different from slip-type rockburst, the high abutment stress should be closely monitoring in predicting strain-type rockburst [20, 21]. Isolated coal pillar mining is one of the typical engineering situations with strain-type rockburst (see Figure 8).

The strain-type rockburst usually occurs in high stress zone of abutment pressure in mining. According to the frictional effect study presented above, the cracks within this zone grow with the mining face advance and the intensity of electromagnetic radiation signal becomes high.

## 4. Application in Predicting Rockburst during Isolated Coal Pillar Extraction

### 4.1. Field Geological Conditions

**Figure 9:**
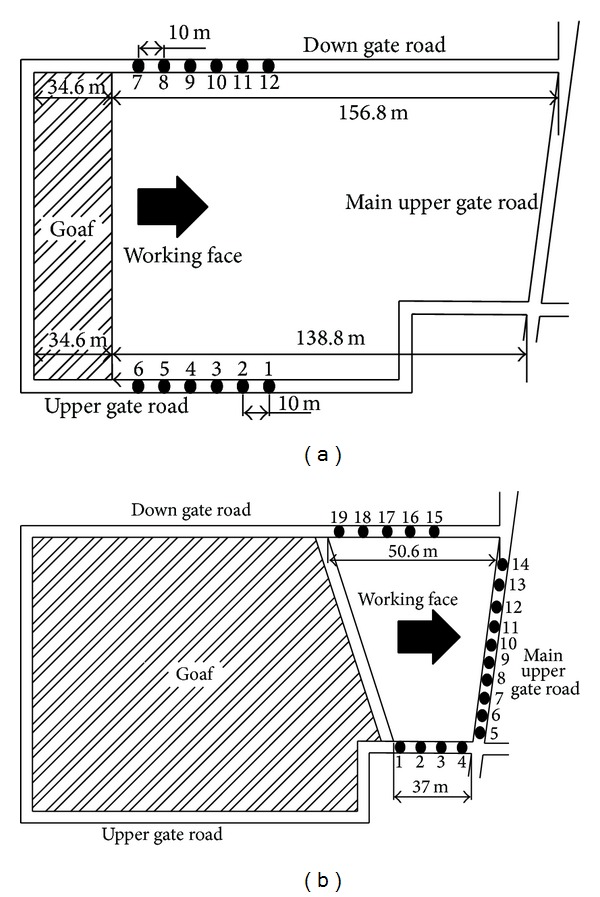
Monitoring disposal around coalface at the (a) early and (b) final stage of extraction.

Field prediction was conducted in an isolated working face located at +250 m level in Mu-cheng-jian coal mine, West Beijing Area, China. The four sides of this working face were already stopped out. The dip length of the working face is 154 m while the strike length is 176 m. At the early stage of mining in this working face, the monitoring positions are set in the upper and down gate road and the interval distance among monitoring points is 10 m. With the advance of extraction, the monitoring points reduced while new monitoring points in the main upper gate road were set as shown in Figure 9.

### 4.2. Monitoring Result Analysis

**Figure 10:**
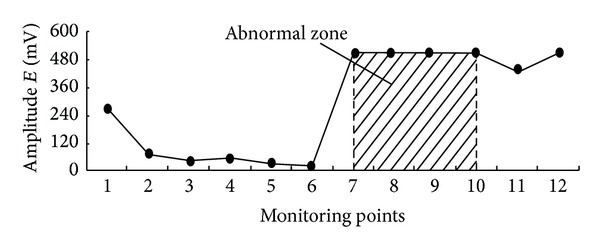
Electromagnetic radiation monitoring results at the early stage of extraction.

**Figure 11:**
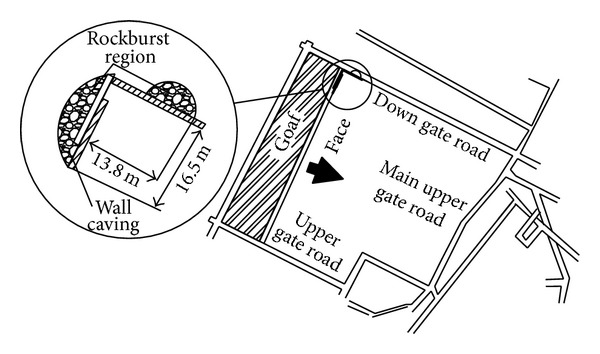
Rockburst region at the early stage of the extraction.

The monitored result of electromagnetic radiation at the early stage of extraction is shown in Figure 10. It can be seen that the amplitude of electromagnetic radiation in down gate road is obviously higher than that in upper gate road, especially in the zone within monitoring points 7-8. That is within the range of 40 m in front of mining face. This indicates that the down gate road is under high stress concentration conditions. When the working face advanced the 30 m, one rockburst occurred.  Figure 11 shows the locality of this rockburst disaster. It can be seen that the actual place of the rockburst is almost the same with that by electromagnetic radiation prediction.

The high stress is due to stress superposition caused by extraction in the four sides of this working face. What is more, there is a stress concentration in advance heading. Such high stress must be relieved in time with the blasting presplitting method. Otherwise it can trigger rockburst disaster.

**Figure 12:**
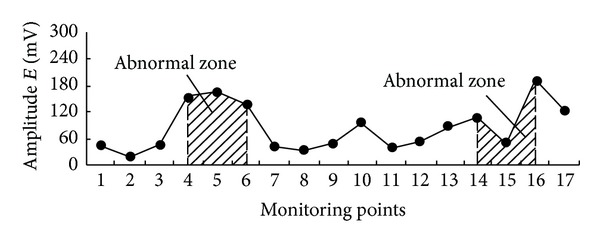
Electromagnetic radiation monitoring results at the final stage of extraction.

**Figure 13:**
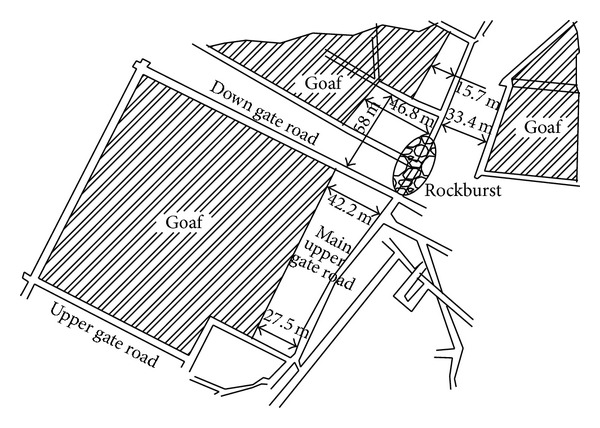
Rockburst region at the ending stage of the extraction.

When advancing to 40 m of upper gate road, the monitored result of electromagnetic radiation is shown in Figure 12. The signal of electromagnetic radiation becomes abnormally high at the intersection of main gate road and two gateways, that is, monitoring points 4–6 and 14–16, indicating the existence of a triangular-shaped high stress concentration zone. Consistent with the prediction, the actual rockburst occurred in the locality as shown Figure 13.

At the ending phase of extraction, the unmined coal pillar becomes smaller and internal stress hence becomes higher. The formation of high stress concentration becomes easier in front abutment pressure zone. Rockburst occurs when the accumulated elastic energy becomes high enough. This is the reason for the occurrence of rockburst when the left unmined zone is of the size 55 × 48 m.

## 5. Conclusions

Numerical simulation based on microparticle flow code shows that uniaxial loading cracked the cohesion among coal particles and caused frictional energy release. Also, it is found that the frictional energy release law is in better accordance with the laboratory experiments of electromagnetic radiation when the friction coefficient decreases linearly with the increase of compressive loading. This provides a fundamental guidance for the application of electromagnetic radiation method to monitor high stress state of surrounding rock during coal mining.

Strain-type rockburst typically occurs in the process of isolated coal pillar extraction. Field applications show that the signals of electromagnetic radiation became obviously abnormal before the occurrence of rockburst, and also the rockburst tends to occur easily at the early and ending stages of isolated coal pillar extraction. The effectiveness of electromagnetic radiation method to predict strain-type rockburst is proved as the actual occurrence zones of rockburst are in good agreement with prediction.

In practice, one of essential conditions for occurrence of strain-type rockburst is high stress concentration and hence the prediction should mainly focus on the monitoring of high stress state. As the electromagnetic radiation monitoring can timely reflect the stress state of coal ore, together with its noncontact and regional monitoring characteristics, it is an effective technique to predict strain-type rockburst.

It is noteworthy that the correlation between the released frictional energy of coal particles and electromagnetic radiation was proposed based on the micronumerical simulation here. Future work will focus on further revelation of prediction mechanism of strain-type rockburst by experimental and theoretical studies.

## Figures and Tables

**Table 1:** Parameters of microstructure model.

Particle radius/mm	Density/kg*·*m^−3^	Young's modulus/GPa	Cohesive strength/MPa
0.2~0.3	2000	5	20
